# Regeneration of injured skeletal muscle in heat shock transcription factor 1-null mice

**DOI:** 10.1002/phy2.71

**Published:** 2013-08-29

**Authors:** Sono Nishizawa, Tomoyuki Koya, Yoshitaka Ohno, Ayumi Goto, Akihiro Ikuita, Miho Suzuki, Tomotaka Ohira, Tatsuro Egawa, Akira Nakai, Takao Sugiura, Yoshinobu Ohira, Toshitada Yoshioka, Moroe Beppu, Katsumasa Goto

**Affiliations:** 1 Department of Orthopaedic Surgery, St. Marianna University School of MedicineKawasaki, Japan; 2 Laboratory of Physiology, School of Health Sciences, Toyohashi SOZO UniversityToyohashi, Japan; 3 Department of Physiology, Graduate School of Health Sciences, Toyohashi SOZO UniversityToyohashi, Japan; 4 Department of Molecular Biology, Graduate School of Medicine, Yamaguchi UniversityUbe, Japan; 5 Department of Exercise and Health Sciences, Yamaguchi UniversityYamaguchi, Japan; 6 Graduate School of Medicine, Osaka UniversityToyonaka, Japan; 7 Hirosaki Gakuin UniversityHirosaki, Japan

**Keywords:** Heat shock proteins, heat shock transcription factor 1, proinflammatory cytokines, skeletal muscle regeneration

## Abstract

The purpose of this study was to investigate a role of heat shock transcription factor 1 (HSF1)-mediated stress response during regeneration of injured soleus muscle by using HSF1-null mice. Cardiotoxin (CTX) was injected into the left muscle of male HSF1-null and wild-type mice under anesthesia with intraperitoneal injection of pentobarbital sodium. Injection of physiological saline was also performed into the right muscle. Soleus muscles were dissected bilaterally 2 and 4 weeks after the injection. The relative weight and fiber cross-sectional area in CTX-injected muscles of HSF1-null, not of wild-type, mice were less than controls with injection of physiological saline 4 weeks after the injury, indicating a slower regeneration. Injury-related increase of Pax7-positive muscle satellite cells in HSF1-null mice was inhibited versus wild-type mice. HSF1-deficiency generally caused decreases in the basal expression levels of heat shock proteins (HSPs). But the mRNA expression levels of HSP25 and HSP90α in HSF1-null mice were enhanced in response to CTX-injection, compared with wild-type mice. Significant up-regulations of proinflammatory cytokines, such as interleukin (IL) -6, IL-1β, and tumor necrosis factor mRNAs, with greater magnitude than in wild-type mice were observed in HSF1-deficient mouse muscle. HSF1 and/or HSF1-mediated stress response may play a key role in the regenerating process of injured skeletal muscle. HSF1 deficiency may depress the regenerating process of injured skeletal muscle via the partial depression of increase in Pax7-positive satellite cells. HSF1-deficiency-associated partial depression of skeletal muscle regeneration might also be attributed to up-regulation of proinflammatory cytokines.

## Introduction

Skeletal muscle has a greater plasticity in response to various extracellular stimuli. Muscle regeneration after injury is highly coordinated process including various cellular responses (Yan et al. 2003). Skeletal muscle regeneration is characterized by two phases: a degenerative phase and a regenerative phase (Chargé and Rudnicki 2004). Early and rapid phase following muscle trauma is characterized by necrosis of damaged myofibers and activation of an inflammation response followed by the secondary phase of the activation of skeletal muscle-specific stem cells, so-called satellite cells (Tidball 2005). Muscle satellite cells play a key role in regeneration of injured skeletal muscle (Seale et al. 2001; Relaix and Zammit 2012). Pathohistological analyses of injured muscle showed that the number of muscle satellite cells increases and those cells differentiate into myotubes and myofibers (Saito and Nonaka 1994). Muscle satellite cells, which express Pax7 in their nuclei, are essential for skeletal muscle regeneration (Seale et al. 2000; Relaix and Zammit 2012). In addition, it was reported that higher level of Pax7-postive satellite cells facilitates the regenerative process of injured muscles (Morioka et al. 2008; Matsuba et al. 2009; Naito et al. 2009), suggesting that the level of Pax7-positive satellite cells may play potential role(s) in skeletal muscle regeneration. Although numerous studies have been performed to elucidate regenerative process of injured skeletal muscle, regulatory mechanisms of skeletal muscle regeneration are not fully elucidated.

Heat shock proteins (HSPs), which act as molecular chaperones, play a part of the tightly regulated systems for maintenance of cellular homeostasis for survival in response to various pathological conditions (Hartl 1996; Fink 1999). HSPs, especially inducible 70 kDa HSP (HSP70, so-called HSP72), as well as HSP25, are induced and protect against cellular stresses via so-called stress response (Welch 1991, 1992; Craig et al. 1993; Morimoto 1993; Miyabara et al. 2006; Hayes et al. 2009). It has been reported that muscle necrosis accompanies the up-regulations of HSPs, such as HSP70 and HSP90 (Bornman et al. 1998; Santoro 2000) in *mdx* mice, which are the murine model for Duchenne muscular dystrophy. Up-regulation of HSP47, which is known as a collagen-specific HSP, suggests that potential fibrosis during skeletal muscle regeneration (Higuchi et al. 2007). However, it is still not known whether heat shock transcription factor 1 (HSF1)-deficiency influences the expression level of HSP47 during skeletal muscle regeneration.

HSFs, which mediate stress response, up-regulate the expression of HSPs via binding to heat shock element located on the up-stream region of HSP genes (Morimoto 1998). Among three HSFs (HSF1, HSF2, and HSF4) in mammals, HSF1 plays a crucial role in inducing HSPs, conferring cytoprotection against various stresses (Zhang et al. 2002; McArdle et al. 2006). However, a physiological role of HSF1-mediated stress response in regeneration of injured skeletal muscle is still not clear.

During the early inflammatory responses to muscle injury, proinflammatory cytokines, such as interleukin-6 (IL-6) and IL-1β, are up-regulated and enhance inflammatory response (Fielding et al. 1993; Tidball 2005). IL-6, IL-1β, and tumor necrosis factor (TNF) are potentially mitogenic for myoblasts, as well as inhibitors of myogenic differentiation (Alvarez et al. 2002; Broussard et al. 2004; Alter et al. 2008). On the other hand, it has been reported that HSF1 suppresses inflammatory genes, including IL-6, through activating transcription factor 3 (ATF3) in cultured embryonic fibroblasts cells (Takii et al. 2010). Although the interactions among HSF1, IL-6, and ATF3 in skeletal muscle cells remains unclear, HSF1 may be a key molecule to regulate regenerative process of injured skeletal muscle involving inflammatory responses. However, there is no report regarding a role of HSF1 in regeneration of injured skeletal muscle. The purpose of this study was to investigate a physiological role of HSF1 gene on skeletal muscle regeneration by using the HSF1-null mice.

## Material and Methods

### Animals

Male HSF1-null and wild-type (ICR) mice with 10–15 weeks of age (*n* = 24) were used as in our previous study (Yasuhara et al. 2011). The experimental procedures were carried out in accordance with the Guide for the Care and Use of Laboratory Animals as adopted and promulgated by the National Institutes of Health (Bethesda, MD) and were approved by the Animal Use Committee at Toyohashi SOZO University. Two or three mice were housed in a cage (20 × 31 cm and 13.5 cm height) in a vivarium room with 1212-h light:dark cycle and with maintained temperature and humidity at ∼23 ± 1 (Mean ± SEM) °C and ∼50%. Solid food and water were provided ad libitum.

### Muscle injury model

Necrosis-regeneration cycle was induced by using intramuscular injection of 0.1 mL cardiotoxin (CTX, 10 μmol/L in physiological saline (PS), Sigma, St. Louis, MO) of Naja naja atra venom. Injection of CTX was performed into the left soleus muscle of mice, using a 27-gauge needle under anesthesia with intraperitoneal injection of pentobarbital sodium as described earlier (Morioka et al. 2008; Matsuba et al. 2009). This procedure for the initiation of necrosis-regeneration was performed carefully to avoid the damage to the nerves and blood vessels, as was suggested elsewhere (Couteaux et al. 1988; Fletcher and Jiang 1993). The same volume of PS was also injected similarly into the right soleus. In this study, there was no significant effect of PS-injection on the analyzed parameters in both types of mice during the entire experimental period. The mice were housed in the same cages for 2–4 weeks.

### Samplings

Soleus muscles were dissected from the both hindlimbs 2 and 4 weeks after CTX- or PS-injection. All muscles were rapidly weighed (wet weight) and divided into three portions cross-sectionally. Then, muscles were frozen in liquid nitrogen, and stored at −80°C until analyzed.

### Muscle protein content

The middle portions of muscles were homogenized in ∼0.4 mL (0.1 mL/mg muscle wet wt) of tissue lysis reagent (CelLytic-MT, Sigma-Aldrich, St.Louis, MO) and completely solubilized by alkaline treatment with 2 N NaOH at 37°C for 1 h (Yasuhara et al. 2011). Protein concentration of the tissue lysate was determined by using Protein assay kit (Bio-Rad, Hercules, CA) and bovine serum albumin (Sigma) as the standard. Total protein content in whole muscle was then calculated.

### Real-time RT-PCR

In this study, the expressions of HSP mRNAs, including HSP25, HSP47, the constitutive cytosolic HSC70, so-called HSP73, and stress-inducible HSP72, and HSP90α, and the expressions of HSF mRNAs including HSF1, HSF2, and HSF4 were assessed by real-time reverse transcription-polymerase chain reaction (real-time RT-PCR). Proinflammatory cytokine mRNAs, such as IL-6, ATF3, IL-1β, and TNF, were also evaluated. Total RNA was extracted from the proximal portion of muscle using the miRNeasy Mini kit (Qiagen, Hiden, Germany), according to the manufacturer's protocol. Samples (∼40 ng of RNA) were reverse-transcribed using the first strand cDNA synthesis kit, according to the manufacturer's instructions (PrimeScript RT Master Mix [Perfect Real Time] for mRNA, Takara Bio, Otsu, Japan). Synthesized cDNA was applied to real-time RT-PCR (Thermal Cycler Dice Real Time System II MRQ, Takara Bio) using Takara SYBR Premix Ex Taq II for mRNA, and analyzed with Takara Thermal Cycler Dice Real Time System Software Ver. 4.00, according to the manufacturer's instructions. The real-time cycle conditions were 95°C for 30 sec followed by 40 cycles at 95°C for 5 sec and at 60°C for 30 sec for mRNA. Specificity was confirmed by electrophoretic analysis of the reaction products and by inclusion of template- or reverse transcriptase-free controls. To normalize the amount of total RNA present in each reaction, glyceraldehyde-3-phosphate dehydrogenase (GAPDH) was used as an internal standard.

The primers were designed by using the Takara Bio Perfect Real Time Support System (Takara Bio). Primers used for detection of mouse cDNA were as follows: HSP25, 5′-TCCCTGGACGTCAACCACTTC-3′ (forward) and 5′-AGAGATGTAGCCATGTTCGTCCTG-3′ (reverse); HSP47, 5′-TGAGGTCACCAAGGATGTGTGGAG-3′ (forward) and 5′-AGGAGCGGGTCACCATGAAG -3′ (reverse); HSC70, 5′-AGCTGCCTGGCATTTGTGTG-3′ (forward) and 5′-GTGCGGTTACCCTGGTCATTG-3′ (reverse); HSP72, 5′-CAAGAACGCGCTCGAATCCTA-3′ (forward) and 5′-TCCTGGCACTTGTCCAGCAC-3′ (reverse); HSP90α, 5′-CCATGCTAACAGGATCTACAGGA-3′ (forward) and 5′-TCTTCAGTTACAGCAGCACTGG-3′ (reverse); HSF1, 5′-ACAGTGTCACCCGGCTGTTG-3′ (forward) and 5′-GACTGCACCAGTGAGATGAGGAA-3′ (reverse); HSF2, 5′-GCAGTGTTGTTCAACATGTGTCAG-3′ (forward) and 5′-AGTTCCCATCCAGGAATGCAAG-3′ (reverse); HSF4, 5′-TGATGGATCTGGACATGGAGTTG-3′(forward) and 5′-CTAGCATGAGTGGAGTTCCCAGTG-3′ (reverse); IL-6, 5′- CCACTTCACAAGTCGGAGGCTTA-3′ (forward) and 5′- GCAAGTGCATCATCGTTGTTCATAC-3′ (reverse); ATF3, 5′- GCTGCTGCCAAGTGTCGAA-3′ (forward) and 5′- CGGTGCAGGTTGAGCATGTATATC-3′ (reverse); IL-1β, 5′- TCCAGGATGAGGACATGAGCAC-3′ (forward) and 5′- GAACGTCACACACCAGCAGGTTA-3′ (reverse); TNF, 5′- TATGGCCCAGACCCTCACA-3′ (forward) and 5′- GGAGTAGACAAGGTACAACCCATC-3′ (reverse); GAPDH, 5′- TGTGTCCGTCGTGGATCTGA-3′ (forward) and 5′- TTGCTGTTGAAGTCGCAGGAG-3′ (reverse).

### Histochemical and immunohistochemical analyses

Serial transverse cryosections (7-μm thick) of frozen distal portion of soleus muscles were cut at −20°C and mounted on the slide glasses. The sections were air-dried and stained to analyze the degree of muscle damage and repair, the cross-sectional area (CSA) of muscle fibers by staining using hematoxylin and eosin (H&E), and the profiles of Pax7-positive nuclei by the standard immunohistochemical technique, respectively (Kojima et al. 2007).

Monoclonal anti-Pax7 antibody (Developmental Studies Hybridoma Bank, Iowa, IA) was used for the detection of muscle satellite cells (Seale et al. 2000). Cross sections were fixed with paraformaldehyde (4%), and then were post fixed in ice-cold methanol. After blocking by using a reagent (1% Roche Blocking Regent; Roche Diagonost, Penzberg, Germany), samples were incubated with the primary antibodies for Pax7 and rabbit polyclonal anti-laminin. Sections were also incubated with the second primary antibodies for Cy3-conjugated antimouse IgG1 (diluted 1: 500; Jackson Immuno Research, West Grove, PA) and for fluorescein isothiocyanate-conjugated anti-rabbit IgG (diluted 1: 500; Sigma). Nuclei were then stained for 15 min in a solution of 4′,6-diamidino-2-phenylindole (DAPI, 0.5 mg/mL; Sigma). The images of muscle sections were incorporated into a personal computer (DP Manager, version 2.2.1.195, Olympus Japan, Tokyo) by using a microscope (IX 81 Olympus Japan). The CSAs of ∼100 fibers from each muscle were analyzed on laminin-stained images by using ImageJ.

### Immunoblotting analyses

Expressions of HSP25, HSP47, HSP60, HSC70, HSP72, and HSP90α proteins were assessed by immunoblotting assay. Proximal portions of the muscles were homogenized in an isolation buffer of tissue lysis reagent (CelLytic-MT, Sigma-Aldrich) with 1 mmol/L Na3VO4, 1 mmol/L phenylmethylsulfonyl fluoride (PMSF) and 1 g/mL leupeptin with glass homogenizer. The homogenates were then sonicated and centrifuged at 15,000 g (4°C for 10 min), and the supernatant was collected.

A part of the supernatant was solubilized in sodium-dodecylsulfate (SDS) sample buffer (30% [vol/vol] glycerol, 5% [vol/vol] 2-mercaptoethanol, 2.3% [wt/vol] SDS, 62.5 mmol/L Tris·HCl, 0.05% [wt/vol] bromophenol blue, and pH 6.8) at a concentration of 0.5 mg of protein per milliliter and boiled for 3 min. The SDS-polyacrylamide gel electrophoresis (PAGE) was carried out on 10 or 12.5% polyacrylamide (bisacrylamide/acrylamide, 1:20 [wt/wt]) slab gel (60 × 85 × 1 mm) containing 0.5% SDS at a constant current of 20 mA for 120 min, as was reported previously (Goto et al. 2004). Equal amounts of protein (20 μg) were loaded on each gel. Molecular weight markers (ECL DualVue Western Blotting Markers, GE Healthcare, Buckinghamshire, UK) were applied to both sides of 14 lanes as the internal controls for transfer process or electrophoresis.

Following SDS-PAGE, proteins were transferred to polyvinylidene difluride membranes (0.2-μm pore size, Bio-Rad) at a constant voltage of 100 V for 60 min at 4°C. The membranes were blocked for 1 h using a blocking buffer: 5% skim milk with 0.1% Tween 20 in Tris-buffered saline (TTBS) with pH 7.5. The membranes were incubated for 1 h with a polyclonal antibody for HSP25 (SPA-801; StressGen, Victoria, BC, Canada), HSC70 (SPA-816; StressGen), HSP72 (SPA-812; StressGen), and then reacted with a secondary antibody (goat anti-rabbit immunoglobulin G conjugate to horseradish peroxidase, Cell Signaling Technology, Danvers, MA) for 1 or 2 h. To detect HSP47 and HSP90, we generated antiserum HSP47 and HSP90 by immunizing rabbit as described previously (Fujimoto et al. 2004; Katsuki et al. 2004). After the final wash, protein bands were visualized using chemiluminescence (ECL Advance Western blotting kit; GE Healthcare), and signal density was measured using Light-Capture (AE-6971) with CS Analyzer Ver. 2.08b (ATTO, Tokyo, Japan). Each sample was investigated in duplicate, at least, to ensure that results were not influenced by loading errors. The densities of GAPDH, (G9545, Sigma-Aldrich) were evaluated to ensure the equal loading. Standard curves were constructed during the preliminary experiments to ensure linearity.

### Statistical analysis

All values were expressed as means ± SEM. Statistical significances for body and muscle weights, protein content, and expression levels of mRNA and protein were tested by using two-way (mice and time for body weight; mice and treatments for other measurements except HSF1 mRNA) analysis of variance (ANOVA). When any significant main effects (factors) and interactions among factors were observed, Turkey-Kramer post hoc test was performed in each effect or among groups. For HSF1 mRNA, data were analyzed using one-way ANOVA followed by Turkey-Kramer post hoc test. The significant level was accepted at *P* < 0.05.

## Results

### Body weight and soleus muscle wet weight

**Figure 1:**
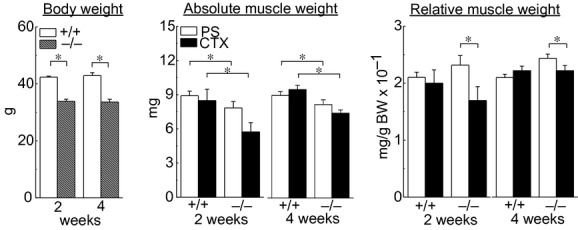
Changes in the body weight and absolute and relative muscle wet weight of heat shock transcription factor (HSF1)-null and wild-type mice 2 and 4 weeks after muscle injury. Relative muscle weight, the muscle weight relative to body weight (BW); +/+ and −/−, wild-type and HSF1-null mice; PS and CTX, physiological saline and cardiotoxin-injected muscle; 2 and 4, 2 and 4 weeks after CTX-injection, respectively. Values are means ± SEM. *n* = 6/group at each time point. *P < 0.05.

Changes in the body weight, absolute muscle wet weight, and relative muscle weight to body weight of HSF1-null and wild-type mice are shown in Figure 1. Even though the body weight in each group was similar between the 2nd and 4th week, that in HSF1-null mice was constantly less than that in wild-type mice (*P* < 0.05). The absolute muscle weight in HSF1-null mice was also constantly less than that in wild-type mice (*P* < 0.05). In wild-type mice, there was no significant change in the relative soleus weight between CTX- and PS-injected groups 2 and 4 weeks after the injection. In HSF1-null mice, on the other hand, CTX-injected soleus had less weight compared with PS-injected muscle in HSF1-null mice 2 weeks after the injection (∼73%, *P* < 0.05). The muscle weight in HSF-null mice increased toward the contralateral control level thereafteHowever the weight was still less than controls 4 weeks after (∼91%, *P* < 0.05).

### Morphology of fibers

**Figure 2:**
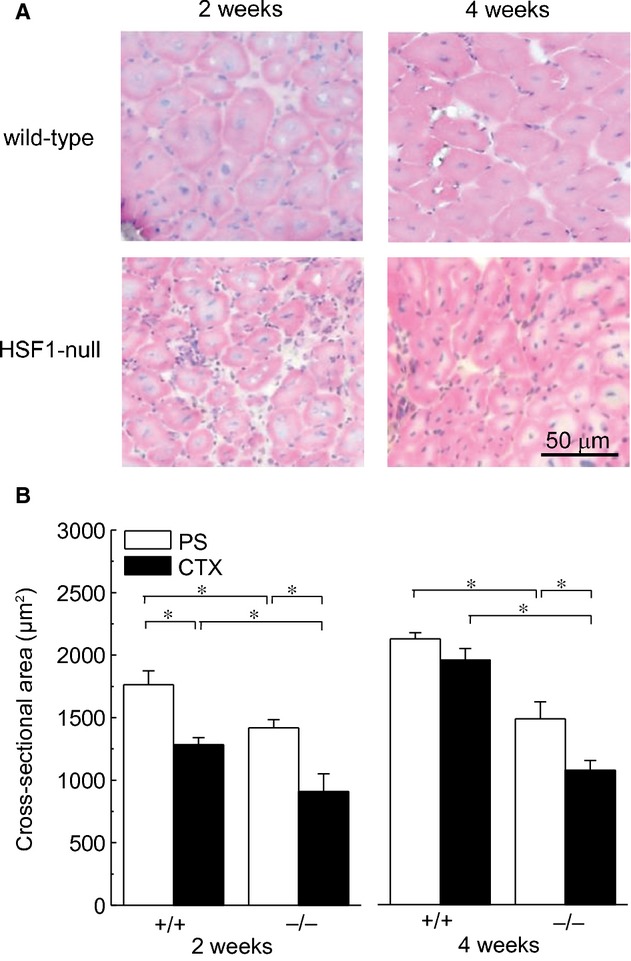
(A) Transverse cryosections of the midbelly region of mouse soleus muscle stained with hematoxylin and eosin. (B) Responses of the mean fiber cross-sectional area. See Figure 1 for other abbreviations. Values are means ± SEM. *n* = 6/group at each time point. *P < 0.05.

The morphological responses of CTX-injected muscles were compared between HSF1-null and wild-type mice 2 and 4 weeks after induction of damage (Fig. 2A). Two weeks after CTX-injection, infiltrating cells and regenerating fibers with central nuclei were observed in the injured muscles of both types of mice. Many inflammatory cells were noted especially in the muscles of HSF-null mice 2 weeks after the CTX-injection. In HSF1-null mice, regenerating fibers with small diameter and central nucleus were seen even after 4 weeks, compared with wild-type mice. It was suggested that HSF1-deficiency delayed the regeneration of injured soleus muscle.

Mean fiber CSAs in wild-type mice were significantly larger than those in HSF1-null mice 2 and 4 weeks after injection of PS or CTX (Fig. 2B, *P* < 0.05). In both wild-type and HSF1-null mice, significant decreases of fiber CSAs were observed 2 weeks after the CTX-injection (*P* < 0.05). Significantly less fiber CSA was still observed in HSF1-null, but not in wild-type, mice 4 weeks after CTX-injection (*P* < 0.05). The mean CSAs in wild-type and HSF1-null mice were ∼92% and ∼73% of the contralateral control level 4 weeks after CTX-injection.

### Pax7-positive nuclei

**Figure 3:**
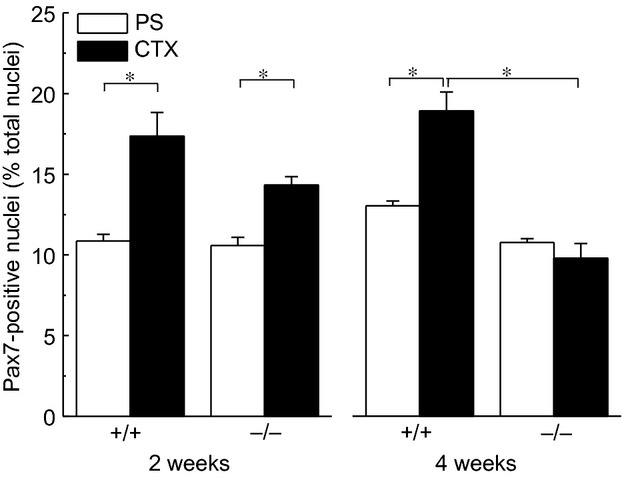
Changes in the proportion of Pax7-positive nuclei relative to total myonuclei during regenerating process of injured skeletal muscle. See Figure 1 for other abbreviations. Values are means ± SEM. *n* = 4–6/group at each time point. *P < 0.05.

Figure 3 shows the changes in the population of Pax7-positive nuclei relative to the total myonuclei during regenerating process of injured skeletal muscle. Relative population of Pax7-positive nuclei in both types of mice was increased by CTX-injection (*P* < 0.05). But that in HSF1-null mice was insignificantly less than that in wild-type mice. Four weeks after CTX-injection, a significantly higher level of population of Pax7-positive nuclei in wild-type, not in HSF1-null mice remained versus controls (*P* < 0.05). Significantly more population of Pax7-positive nuclei was noted in wild-type than HSF1-null mice 4 weeks after CTX-injection (*P* < 0.05).

### HSF genes

**Figure 4:**
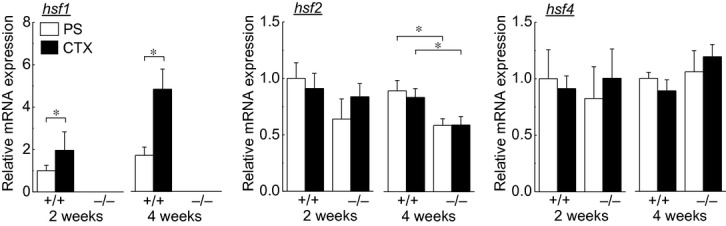
Changes in the mean expression levels of heat shock transcription factor (HSF) 1, HSF2, and HSF4 mRNAs in soleus muscle during the experimental period. *hsf1*, HSF1 mRNA; *hsf2*, HSF2 mRNA; *hsf4*, HSF4 mRNA. See Figure 1 for other abbreviations. Values are means ± SEM. *n* = 5–6/group at each time point. *P < 0.05.

Changes in the mean expression levels of HSF1, HSF2, and HSF4 mRNAs in soleus muscle are shown in Figure 4. HSF1 gene was not detected in HSF1-null mice. In wild-type mice, the up-regulation of HSF1 mRNA was observed 2 weeks after CTX-injection (∼+96%, *P* < 0.05). Higher level of HSF1 mRNA in CTX-injected muscle, compared to the contralateral control level, was also observed 4 weeks after CTX-injection (∼+282%, *P* < 0.05). Expression level of HSF2 mRNA in HSF1-null mice was significantly less than wild-type mice 4 but not 2, weeks after injection of PS or CTX (*P* < 0.05). There was no significant difference in HSF4 mRNA of both types of mice during the experimental period.

### HSPs expressions

**Figure 5:**
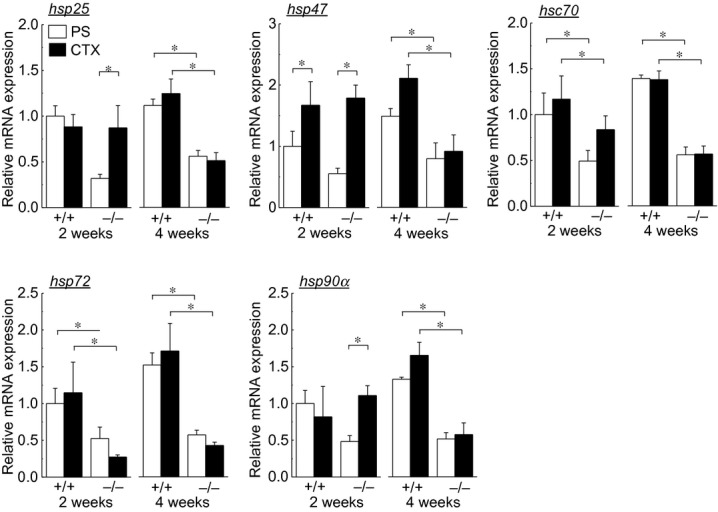
Changes in mRNA expressions of heat shock protein (HSP) 25, HSP47, HSC70, HSP72, and HSP90. *hsp25*, HSP25 mRNA; *hsp47*, HSP47 mRNA; *hsp60*, HSP60 mRNA; *hsc70*, HSC70 mRNA; *hsp72*, HSP72 mRNA; *hsp90*, HSP90 mRNA. See Figure 1 for other abbreviations. Values are means ± SEM. *n* = 6/group at each time point. *P < 0.05.

The expression levels of HSPs were evaluated by real-time RT-PCR (at the transcriptional level) and immunoblotting assay (at protein level) in this study. Changes in mRNA expressions of HSP25, HSP47, HSC70, HSP72, and HSP90α are shown in Figure 5. Absence of HSF1 gene caused the lower expression levels of HSP25 (∼−59%, *P* > 0.05), HSP47 (∼−45%, *P* > 0.05), HSC70 (∼−51%, *P* < 0.05), HSP72 (∼−48%, *P* < 0.05), and HSP90α (∼−52%, *P* > 0.05) in the PS-injected control muscles 2 week after the injection.

In HSF1-null, not wild-type, mice, the level of HSP25 mRNA increased 2 weeks after CTX-injection (∼+210%, *P* < 0.05). The HSP47 mRNA expression was significantly up-regulated in both wild-type and HSF1-null mice 2 weeks after CTX-injection (∼+67% and 223%, respectively). The mRNA expressions of HSP90α in HSF1-null, not wild-type, mice also increased 2 weeks after CTX-injection significantly (∼+160%). These levels in HSF1-null mice decreased and became identical to their controls at the 4th week. The CTX-related up-regulation was not induced in the mRNA expression of HSC70 and HSP72 in HSF1-null mice. And these levels at the 4th week were significantly less than those in wild-type mice.

**Figure 6:**
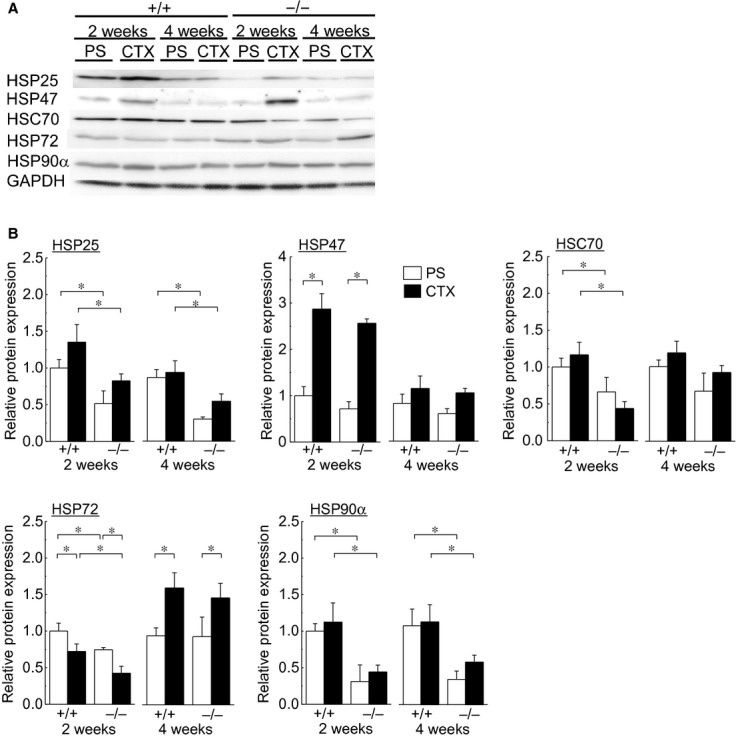
Expressions of heat shock proteins (HSPs) and heat shock cognate protein (HSC) in soleus muscle. (A) Representative expression patterns of HSP25, HSP47, HSP60, HSC70, HSP72, HSP90α, and glyceraldehyde-3-phosphate dehydrogenase (GAPDH). (B) Changes in the mean protein expression levels of HSP25, HSP47, HSP60, HSP70, HSP72, and HSP90α. See Figure 1 for other abbreviations. Values are means ± SEM. *n* = 6/group at each time point. *P < 0.05.

Representative expression patterns of HSP25, HSP47, HSC70, HSP72, HSP90α, and GAPDH are shown in Figure 6A. Two weeks after the PS-injection, the protein expression levels of HSP25 (∼−49%, *P* < 0.05), HSP47 (∼−28%, *P* > 0.05), HSC70 (∼−34%, *P* < 0.05), HSP72 (∼−26%, *P* < 0.05), and HSP90α (∼−69%, *P* < 0.05) of PS-injected control soleus muscle in HSF1-null mice were lower than those in wild-type mice.

Protein expression levels of HSP25, HSC70, HSP72, and HSP90α in HSF1-null mice 2 weeks after CTX-injection were significantly less than in wild-type mice (Fig. 6B, *P* < 0.05). Significant effects of CTX-injection on these levels were not observed in HSP25, HSC70, and HSP90α at the 2nd week. However, the expression of HSP72 was lowered in both wild-type and HSF1-null mice (∼−28% and ∼−43%, respectively, *P* < 0.05). The differences in HSP25 and HSP90α expression seen between two species remained at the 4th week. However, the differences in HSC70 and HSP72 between the two types of mice disappeared after 4 weeks. The levels of HSC70 became identical between two species, and those of HSP72 in the muscles with CTX-injection were even elevated in both wild-type (∼+69%) and HSF1-null (∼+57%) mice, compared to the contralateral controls.

There was no significant difference in the expression level of HSP47 protein between two types of mice. Greater increases in the HSP47 protein content in both wild-type (∼+187%, *P* < 0.05) and HSF1-null mice (∼+258%, *P* < 0.05) were observed 2 weeks after CTX-injection. However, these levels in both types of mice returned to the PS-injected contralateral controls level at the 4th week.

### Proinflammatory cytokines

**Figure 7:**
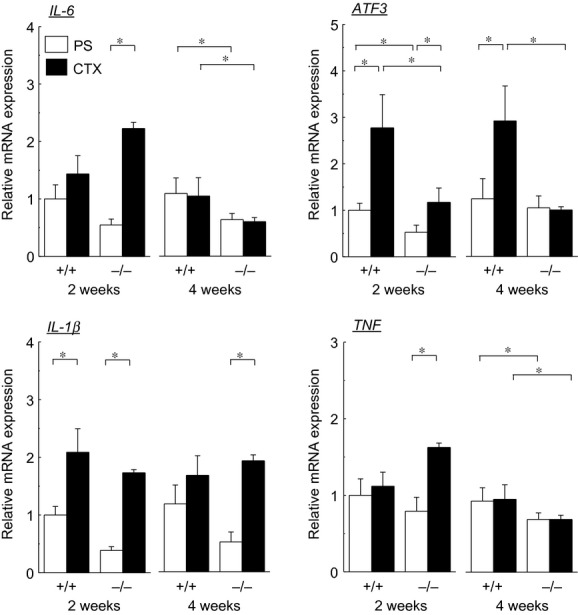
Changes in the mean mRNA expression levels of proinflammatory cytokines in soleus muscle. *IL-6*, interleukin-6; *ATF3*, activating transcription factor 3; *IL*-*1*β, interleukin1β; *TNF*, tumor necrosis factor. See Figure 1 for other abbreviations. Values are means ± SEM. *n* = 6/group at each time point. *P < 0.05.

Changes in the mean mRNA expression levels of proinflammatory cytokines, IL-6, ATF3, IL-1β, and TNF, in soleus muscle are shown in Figure 7. The expression level of IL-6 mRNA in the PS-injected control muscles of wild-type mice was greater than HSF1-null mice at 4th week (*P* < 0.05) time point. Up-regulation of IL-6 mRNA was observed in HSF1-null mice 2 weeks after CTX-injection (∼+307%, *P* < 0.05), although that in wild-type mice was minor (∼+43%, *P* > 0.05). However, these levels returned to the control levels at the 4th week.

The expression level of ATF3 mRNA in the PS-injected control muscles of HSF1-null mice was significantly lower than that in wild-type mice at the 2nd week (*P* < 0.05). Although the levels were increased by injection of CTX in both types of mice 2 weeks after induction of injury (*P* < 0.05), the response in wild-type (∼+177%) was greater than in HSF1-null mice (∼+122%, *P* < 0.05). However, the expression level of ATF3 mRNAs in HSF1-null, not in wild-type, mice was normalized and became significantly less than that in wild-type mice 4 weeks after (*P* < 0.05).

Significant differences were not observed in IL-1β mRNA expression between two species. Up-regulation of IL-1β was induced in response to CTX-injection in both types of mice (*P* < 0.05). These levels remained higher than control even after 4 weeks, although statistical significance was not obtained in the mean level in wild-type mice. Significant up-regulation of TNF mRNA was observed in HSF1-null, not in wild-type, mice 2 weeks after CTX-injection (*P* < 0.05). The mean levels in HSF1-null mice at the 4th week were less than those in wild-type mice regardless of CTX-injection (*P* < 0.05).

## Discussion

This study showed that absence of HSF1 partially delayed skeletal muscle regeneration following CTX-injection-associated muscle injury. This is the first report showing the negative effects of HSF1-deficiency on the regeneration of injured muscle. Population of Pax7-positive muscle satellite cells, which play a key role in skeletal muscle regeneration, in HSF1-null mice was less than that in wild-type mice 4 weeks after the CTX-injection. HSF1-deficiency caused decreases in the expression levels of HSPs (mRNAs of HSC70 and HSP72, and proteins of HSP25, HSC70, HSP72, and HSP90α). The responses of HSP25 and HSP90α mRNAs expressions in HSF1-null mice to CTX-injection-induced muscle injury were enhanced, compared with wild-type mice. CTX-injection-associated up-regulated mRNA expressions of proinflammatory cytokines, such as IL-6 and TNF, were also stimulated by HSF1-decifiency. On the other hand, CTX-injection-associated up-regulation of ATF3 was depressed by the absence of HSF1 gene.

### Effects of HSF1-deficiency on skeletal muscle regeneration

This study demonstrated that HSF1-deficiency delayed the regeneration of injured soleus muscle. Population of Pax7-positive satellite cells increases in muscle following CTX-injection-associated injury (Matsuba et al. 2009). In this study, HSF1-deficiency did not affect the population level of Pax7-positive satellite cells in the control muscles. However, CTX-injection-associated increases of Pax7-positive satellite cells were inhibited in HSF1-null mice. Although the percent population of Pax7-positive cells was also increased 2 weeks after CTX-injection, the magnitude was minor relative to that in wild-type mice (∼85%, *P* < 0.05). And the injury-related increase in population was normalized 4 weeks after. Therefore, it was speculated that HSF1 may play a part in the regulation of proliferative potential of muscle satellite cells following muscle injury.

Mechanical overloading and application of heat stress have stimulating effects on the regenerative potential of injured skeletal muscle (Kojima et al. 2007; Morioka et al. 2008) and up-regulations of HSPs, such as HSP25, HSP72, and HSP90 (Oishi et al. 2003; Lawler et al. 2006; Locke 2008; Huey et al. 2010). However, it is still not clear whether stress-related facilitation of skeletal muscle regeneration is attributed to the chaperonic action of HSPs or stress-associated stimulation of protein synthesis. In this study, absence of HSF1 caused to depress the protein expression of HSP25, HSC70, HSP72, and HSP90α. These results were consistent with those of the previous study (Yasuhara et al. 2011). However, there is still no report regarding the effects of lower expression levels of HSPs on the regeneration of injured skeletal muscle. Recently, it has been reported that loss of inducible HSP70 impaired muscle regeneration (Senf et al. 2013). Lower protein expression levels of HSPs, such as HSP25, HSC70, HSP72, and HSP90α, may be one of the causes that delay the regeneration of injured skeletal muscle.

### Possible effects of HSF1 on proinflammatory cytokines during skeletal muscle regeneration

Up-regulation of proinflammatory cytokines was observed following soleus muscle injury in this study at mRNA levels. Up-regulations of IL-6 (at the 2nd week), IL-1β (at the 4th week) and TNF (at the 2nd week) mRNAs during regeneration were observed, but not in wild-type mice. IL-6, IL-1β, and TNF have been reported to inhibit myogenic differentiation (Alvarez et al. 2002; Broussard et al. 2004; Alter et al. 2008). HSF1-deficiency-associated depression of skeletal muscle regeneration may be attributed to the up-regulation of proinflammatory cytokines, such as IL-6, IL-1β, and TNF.

However, the molecular mechanisms of HSF1-deficiency-associated up-regulation of proinflammatory cytokines in injured skeletal muscle are still not clear. However, one possibility might be a direct action of HSF1 on ATF3. HSF1 inhibits IL-6 expression through activation of ATF3 (Takii et al. 2010). Although up-regulation of ATF3 mRNA, regardless of HSF1 expression, was observed 2 weeks after CTX-injection in this study, HSF1-deficiency depressed the magnitude of increase in the expression level of ATF3 compared with wild-type mice. Therefore, higher expression of IL-6 in HSF1-null mice may be attributed to the lower mRNA expression level of ATF3.

### Effects of HSF1-deficiency on the expression level of HSPs following skeletal muscle injury

In this study, up-regulations of HSPs were observed in soleus muscles of both wild-type and HSF1-deficient mice, although it is reported that the up-regulation of HSPs in mammalian skeletal muscle is mediated by HSF1 (Zhang et al. 2002; McArdle et al. 2006). The up-regulations of HSPs in HSF1-null mice were not accompanied by up-regulation of HSF2 and HSF4 mRNAs (Fig. 4). Yasuhara et al. (2011) reported that up-regulation of HSPs during mechanical-load-associated re-growth of soleus muscle in HSF1-null mice was accompanied by up-regulation of HSF2 and HSF4 mRNAs. However, we have no clear explanation for the up-regulations of HSPs in regenerating soleus muscle of HSF1-null mice yet. Posttranscriptional regulation of HSP expressions, such as noncoding microRNA, has been suggested (Ogata et al. 2009). Unknown regulatory system(s) might exist in skeletal muscle cells.

In conclusion, HSF1-deficiency retarded the regeneration of injured mouse soleus muscle. Injury-related-increase in Pax7-positive satellite cells was partially depressed by absence of HSF1. HSF1-deficiency stimulated the injury-related increase in proinflammatory cytokines, such as IL-6, IL-1β, and TNF, which act as the inhibitors for myogenic differentiation and then inhibit the muscle regeneration. HSF1 and/or HSF1-mediated stress response may play a key role in the regeneration of injured skeletal muscle.
